# Comparative Renal Effects of Continuous Infusion Versus Intermittent Bolus Dosing of IV Loop Diuretics in Acute Decompensated Heart Failure: A Meta-Analysis

**DOI:** 10.7759/cureus.88953

**Published:** 2025-07-29

**Authors:** Abbeykeith Kugasenanchettiar, Kowshijan Vasanthan, Arkaprabha Saha, Oltiana Lakra

**Affiliations:** 1 General Internal Medicine, Ealing Hospital, Southall, GBR; 2 General Internal Medicine, George Eliot Hospital, Nuneaton, GBR; 3 General Internal Medicine, East Surrey Hospital, Redhill, GBR; 4 Cardiology, London North West University Healthcare NHS Trust, Harrow, GBR

**Keywords:** acute decompensated heart failure (adhf), continuous infusion loop diuretic, decompensated heart failure, diuretic therapy, heart failure hospitalization

## Abstract

IV loop diuretics remain the cornerstone of treatment for acute decompensated heart failure (ADHF). Although previous meta-analyses have compared continuous infusion and intermittent bolus dosing of IV loop diuretics, their respective renal effects remain unclear. Given the prognostic significance of worsening renal function (WRF) or acute kidney injury in ADHF, evaluating the renal safety of different diuretic regimens is essential. We conducted a systematic search of the PubMed database and performed a meta-analysis of randomized controlled trials (RCTs) comparing both diuretic strategies. The primary outcome was WRF, while secondary outcomes included increases in serum creatinine (sCr), sCr levels at discharge, discharge blood urea nitrogen (BUN) levels, and length of hospitalization. A post hoc trial sequential analysis (TSA) was also conducted to assess the adequacy of the current pooled evidence. A total of 11 RCTs were included. There was no statistically significant difference in the incidence of WRF between continuous infusion and intermittent bolus dosing (RR 1.12; 95% CI, 0.86 to 1.48; I² = 0.00%). Similarly, no significant differences were found in secondary outcomes: increase in sCr (mean difference (MD) 0.24 mg/dL; 95% CI, -0.17 to 0.66 mg/dL; I² = 98.7%), sCr at discharge (MD 0.33 mg/dL; 95% CI, -0.13 to 0.80 mg/dL; I² = 69.6%), discharge BUN levels (MD 6.57 mg/dL; 95% CI, -7.93 to 21.80 mg/dL; I² = 78.7%), and length of hospitalization (MD -0.50 days; 95% CI, -2.75 to 1.76 days; I² = 93.0%). The post hoc TSA revealed that the current evidence base is underpowered and inconclusive. Limited heterogeneity (I² = 0%) was observed among studies reporting WRF, indicating consistency in this primary outcome. However, the high I² values and wide CIs in the secondary outcomes reflect imprecise effect estimates, thereby limiting the clinical certainty of these findings. The TSA calculated a required information size of 3,342 participants, whereas the accrued information size in this meta-analysis was only 693 participants. This discrepancy underscores the potential for a type II error and reinforces the conclusion that current evidence remains insufficient to draw definitive conclusions. Overall, continuous infusion of loop diuretics does not appear to provide a significant renal advantage over intermittent bolus administration. The substantial evidence gap highlights the need for larger, high-quality RCTs powered to detect clinically meaningful renal outcomes. This study represents the first meta-analysis to prioritize renal endpoints and incorporate TSA in comparing these two diuretic strategies.

## Introduction and background

Congestive heart failure refers to a clinical syndrome characterized by an impairment in the ability of the heart to pump sufficient blood to meet the metabolic demands of the body. Acute decompensated heart failure (ADHF) represents an abrupt exacerbation of this condition, leading to peripheral volume overload, dyspnea, and elevated cardiac filling pressures. In the United States, ADHF imposes a significant healthcare burden, accounting for 1.76 million hospitalizations per annum [1].

International guidelines recommend the use of IV loop diuretics to manage the vascular and peripheral congestion associated with ADHF. According to the 2021 European Society of Cardiology guideline and the 2022 joint guideline from the American Heart Association, the American College of Cardiology, and the Heart Failure Society of America, prompt initiation of IV loop diuretics is recommended to encourage decongestion of patients presenting with ADHF [2,3].

Loop diuretics, of which furosemide is the most widely used, inhibit the sodium-potassium-chloride (Na-K-Cl) cotransporter located on the apical membrane of cells lining the thick ascending limb of the loop of Henle [4]. In doing so, these agents produce potent natriuretic and diuretic effects, reducing intravascular and interstitial fluid volumes [4]. These decongestive functions reduce cardiac preload and ameliorate pulmonary congestion, resulting in profound symptomatic benefit.

The reductions in effective circulating volume created by IV diuretic administration may trigger compensatory neurohormonal responses through activation of the renin-angiotensin-aldosterone system (RAAS) and the sympathetic nervous system [4,5]. Excessive activation of the RAAS can result in higher levels of angiotensin II, resulting in afferent renal arteriolar vasoconstriction, glomerular hypoperfusion, and nephronal ischemia [6]. Similarly, activation of the sympathetic nervous system leads to stimulation of α1-adrenergic receptors in the renal vasculature, leading to constriction of afferent and efferent arteriolar beds [7]. This further exacerbates glomerular hypoperfusion and, in conjunction with the RAAS, contributes to prerenal azotemia, acute kidney injury (AKI), and global renal dysfunction. These physiological effects underscore the need for judicious use of IV loop diuretics in patients with ADHF and may be especially pronounced in patients with nephrological comorbidities, such as chronic kidney disease.

Continuous infusions of loop diuretics theoretically mitigate fluctuations in plasma diuretic concentrations, potentially reducing neurohumoral and sympathetic nervous system activation. Conversely, intermittent bolus administration may create significant peak-trough fluctuations in plasma diuretic concentration, intensifying these compensatory mechanisms. Although physiologically plausible, this rationale remains theoretical in nature and is not robustly supported by clinical data.

In a previous meta-analysis, the in-hospital mortality rate associated with AKI was 21% and rose to 46% in patients requiring renal replacement therapy [8]. The development of AKI has further been associated with increased readmission rates for any cause (HR 1.62; 95% CI 1.60-1.65) and for heart failure specifically (HR 2.81, 95% CI 2.66-2.97) [9]. Furthermore, in the context of ADHF, worsening renal function (WRF) has been independently associated with a 1.35-fold increase in mortality, compared to those without WRF [10]. The severity of WRF has further been positively correlated with mortality in a previous meta-analysis [11]. Given these strong mortality and morbidity risks, it is prudent to minimize renal impairment when using IV diuretics to treat ADHF. Accordingly, this study reports on the incidence of WRF/AKI in patients receiving continuous infusions and intermittent bolus injections of loop diuretics as a primary outcome.

While indispensable in enabling rapid decongestion of volume-overloaded patients, the large doses of IV loop diuretics that are often required to treat ADHF can precipitate renal dysfunction and increase mortality. Despite these risks, current international guidelines do not make explicit recommendations as to whether IV loop diuretics should be administered as continuous infusions or as intermittent boluses, leaving such decisions to the discretion of treating physicians. Such discretion contributes to real-world variability in diuretic administration practices, which may also be affected by other clinician-specific and institutional factors.

Pharmacokinetically, continuous infusions enable steady plasma diuretic concentrations to be reached and reduce the fluctuations in plasma diuretic concentration noted with intermittent bolus administration [12]. In doing so, continuous infusions are theorized to result in reduced neurohumoral and sympathetic nervous system activation, leading to a lower renal burden and a reduced risk of renal injury. Hence, where renal safety is a key consideration in the inpatient management of ADHF, physicians may choose to administer IV diuretics as continuous infusions, as renal insults portend poor outcomes as detailed above.

Conversely, intermittent boluses may be favored in other settings. From a resource-utilization perspective, intermittent bolus administration of IV diuretics is easier and does not require infusion pumps in the same way as continuous infusions, reducing the burden on healthcare personnel. Intermittent boluses may also allow nephrons to benefit from “drug-free” intervals, possibly leading to renal and hemodynamic recovery.

Multiple randomized controlled trials (RCTs) have been performed to compare continuous IV infusions of loop diuretics with intermittent bolus administration. The results of these trials have further been pooled in meta-analyses [13-15]; however, these reviews compared the safety and efficacy endpoints, such as symptomatic relief, mortality, and length of hospitalization, rather than renal outcomes.

Whereas earlier systematic reviews and meta-analyses focused on symptom relief, hemodynamic responses, and morbidity [13-15], the present meta-analysis concentrates on renal outcomes to determine whether this hypothesized renoprotective effect translates into clinical benefit. Previous meta-analyses did not focus explicitly on the incidence of WRF or AKI, although Ng and Yap [15] did note no difference in the incidence of raised creatinine between patients receiving continuous infusions and intermittent boluses of loop diuretics. This meta-analysis further contributes to that of previous works, including Karedath et al. [14], by including two studies not considered by this previous meta-analysis [16,17]. To our knowledge, this is also the first meta-analysis to use a trial sequential analysis (TSA) to determine whether the current evidence is sufficient to support its clinical conclusions. Although previous meta-analyses have suggested that further RCTs are required on this matter, this meta-analysis uses a TSA to evaluate the robustness of the present evidence and quantify the evidence that may be required to draw robust conclusions.

Recognizing the prognostic effect of renal impairment in ADHF and the ongoing uncertainty regarding the optimal mode of IV diuretic administration, this meta-analysis compares the renal outcomes associated with continuous infusion and intermittent bolus administration of IV loop diuretics in patients hospitalized with ADHF.

## Review

Methods

This meta-analysis was conducted in accordance with the methodology of the Cochrane Collaboration [18] and Preferred Reporting Items for Systematic reviews and Meta-Analyses (PRISMA) guidelines [19].

Eligibility Criteria

All RCTs comparing continuous versus intermittent IV loop diuretics in adult human patients with ADHF were considered for inclusion, and no data criteria were applied for inclusion. Case reports, observational studies, retrospective studies, nonrandomized trials, and randomized crossover trials were excluded. Studies of patients with acute heart failure of non-heart failure origin were further excluded. Owing to the lack of translation facilities, only studies published in the English language were included. Articles from the grey literature and conference abstracts were further excluded.

The primary outcome of interest was WRF, which could be defined by the study authors, but, in the absence of any such definition, was defined per KDIGO criteria for stage 1 AKI [20]. The secondary outcomes of interest were an increase in serum creatinine (sCr), sCr at discharge, serum blood urea nitrogen (BUN) at discharge, and length of hospitalization.

Search Strategy and Selection

Two study authors independently searched the PubMed database for studies published from the inception of the database to April 2025 using a standardized search strategy that included the key terms “heart failure”, “diuretics”, “continuous”, “intravenous”, “intermittent”, and “renal function”, as well as their synonyms and subheadings. The references of previous meta-analyses were also hand-searched for additional trials [14,15,21-23]. The titles and abstracts of studies were independently screened by two study authors, and relevant studies were selected following full-text evaluation. Any disagreements were resolved through discussion and the involvement of the third study author.

Data Extraction

Data from eligible studies were independently extracted using a standardized data extraction form. Examples of information extracted from studies included (1) study characteristics; (2) dosages and types of loop diuretic used; (3) outcome measures; and (4) sample sizes.

Risk of Bias

The risk of bias of each study was independently assessed by two study authors using the Cochrane Risk of Bias 2 tool [24]. Any discrepancies were resolved through discussion and the involvement of the third author.

Statistical Analysis

Data from included trials were collated and analyzed in RStudio. For dichotomous outcomes (WRF), an RR was calculated. For continuous outcomes (increase in sCr, sCr at discharge, serum BUN at discharge, and length of hospitalization), a mean difference (MD) was calculated. 95% CIs were calculated for each summary measure, and a p-value of less than 0.05 was deemed to indicate statistical significance. Statistical heterogeneity was further calculated using I² and Q values. Given the observed heterogeneity between study populations during a preliminary search for this meta-analysis, random-effects models were used in this meta-analysis.

Results

Study Selection

**Figure 1:**
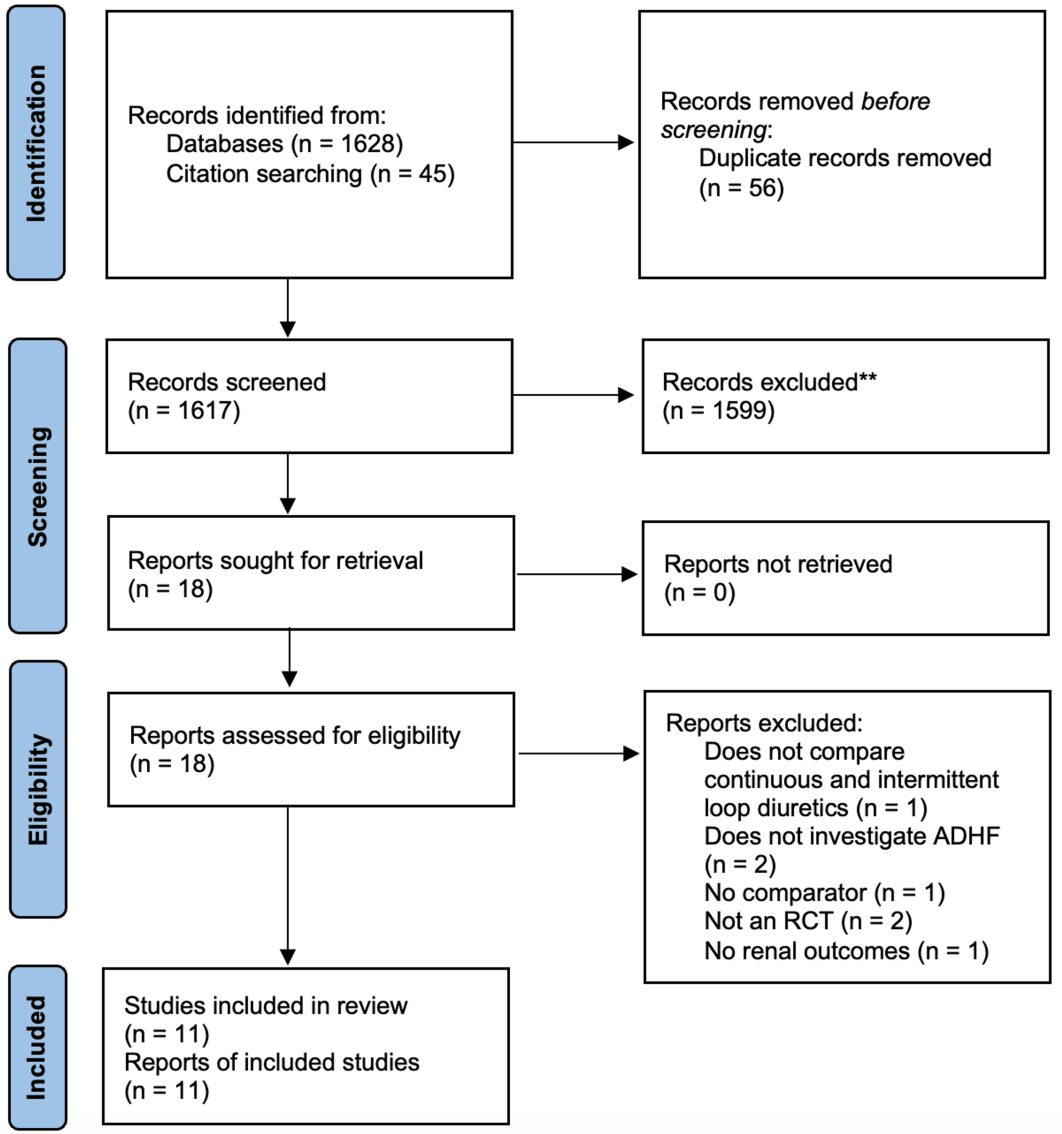
PRISMA flow diagram ADHF, acute decompensated heart failure; PRISMA, Preferred Reporting Items for Systematic reviews and Meta-Analyses; RCT, randomized controlled trial

The search strategies described above yielded 1628 records. Screening the reference lists of other meta-analyses [14,15,21-23] yielded a further 45 records, leading to 1673 records in total. Following the removal of duplicate records, a total of 1617 records were subjected to title and abstract screening. Such screening resulted in 18 records being subject to full-text screening. Following full-text screening, 11 studies were included in this meta-analysis, and seven studies were excluded for various reasons. The results of the literature search and study selection process are depicted in the PRISMA flowchart in Figure 1.

Study Characteristics

Table 1 presents the key characteristics of 11 studies included in the present meta-analysis.

**Table 1:** Characteristics of included studies ADHF, acute decompensated heart failure; eGFR, estimated glomerular filtration rate; RCT, randomized controlled trial

Author	Year	Design	Key inclusion criteria	Details of loop diuretic administration	Number of participants	Mean age of participants (years)	Percentage of male participants (%)	Mean left ventricular ejection fraction (%)	Baseline creatinine concentration (mg dL⁻¹)	Setting
Frea et al. [16]	2020	Double-blind, double-dummy, single-center, RCT	Patients with ADHF, ejection fraction ≤30%, high risk of diuretic resistance	Continuous: 120-240 mg furosemide every 24 hours (mean = 154.5 mg)	40	63	93	19.4	1.7	Italy
				Intermittent: 120-240 mg furosemide every 24 hours (mean = 216 mg)	40	59	88	19.2	1.8	
Khan et al. [17]	2024	Open-label, single-center, RCT	Patients with ADHF	Continuous: 160 mg furosemide over 16 hours	420	72	50	45	1	Pakistan
				Intermittent: 80 mg furosemide boluses twice daily	479	71	64	41	1.1	
Allen et al. [25]	2010	Open-label, single-center, RCT	Patients with ADHF	Continuous: 162 (±52) mg furosemide every 24 hours	20	61	85	31	1.8	USA
				Intermittent: 162 (±48) mg furosemide every 24 hours, as twice-daily boluses	20	58	43	39	2.1	
Felker et al. [26]	2011	Double-blind, multi-center, RCT	Patients with ADHF	Continuous: “Low” dose of furosemide (IV dose equivalent to total oral diuretic doses, in furosemide equivalents) or “high” dose of furosemide (2.5 times daily oral loop diuretic dose) as continuous infusions	152	66	73	35	1.5	Canada and the USA
				Intermittent: “Low” dose of furosemide (IV dose equivalent to total oral diuretic doses, in furosemide equivalents) or “high” dose of furosemide (2.5 times daily oral loop diuretic dose), as boluses every 12 hours	156	66	74	35	1.5	
Jaya Shree et al. [27]	2021	Open-label, single-center, randomized trial	Patients with volume overload, ejection fraction <40%	Continuous: 2-3 mg of furosemide per hour	28	69	50	33	1.7	India
				Intermittent: 40 mg furosemide every eight hours	28	63	64	36	1.2	
Llorens et al. [28]	2014	Open-label, multi-center, RCT	Patients with ADHF	Continuous: 10 mg furosemide per hour over 24 hours	36	82	33	No data provided	1	Spain
				Intermittent: 20 mg furosemide boluses every six to eight hours	73	83	34	No data provided	1.1	
Palazzuoli et al. [29]	2014	Open-label, double-blind, single-center, RCT	Patients with ADHF, ejection fraction <45%	Continuous: Titrated from 80 ± 20 mg to 250 ± 40 mg over 24 hours	43	80	44	34	1.8	Italy
				Intermittent: Titrated from 80 ± 20 mg to 250 ± 40 mg twice daily	39	79	54	36	1.3	
Ragab et al. [30]	2018	Single-center, randomized trial	Patients with volume overload	Continuous: 5 mg of furosemide per hour	20	54	65	38	1.9	Egypt
				Intermittent: 40 mg furosemide boluses every eight hours	20	57	55	37	1.7	
Shah et al. [31]	2014	Single-center, randomized trial	Patients with one symptom and sign of heart failure	Continuous: 100 mg of furosemide over 24 hours	30	59	77	33	1.4	India
				Intermittent: 50 mg of furosemide twice daily	30	59	77	33	1.4	
Yayla et al. [32]	2015	Single-center, randomized trial	Patients with ADHF	Continuous: 160 mg of furosemide over 16 hours	15	65	53	41	1.1	Türkiye
				Intermittent: 80 mg of furosemide twice daily	14	72	50	45	0.9	
Zheng et al. [33]	2021	Open-label, single-center, randomized trial	Patients with ADHF and eGFR between 15.0 and 44.9 mL/min/1.73 m²	Continuous: 160 mg or 200 mg of furosemide over six hours	42	66	67	56	2.3	China
				Intermittent: 160 mg or 200 mg of furosemide as once-daily boluses	39	67	64	59	2.3	

Design

All 11 studies were RCTs. Three studies were double-blind studies [16,26,29], and one of these three studies was a double-blind, double-dummy trial [16]. The remaining eight trials were a mixture of open-label trials, single-blind trials, or trials with unclear detail about blinding [17,25,27,28,30-33]. Only one study [26] was carried out across multiple countries, albeit limited to North America. This is an important consideration, as it may limit the geographical generalizability of findings and applicability across different healthcare systems.

Participant Characteristics

A total of 1784 participants were enrolled across the 11 studies included in this meta-analysis, with 846 participants receiving continuous furosemide infusions and 938 participants receiving intermittent IV boluses. The range of sample sizes (combining continuous and intermittent bolus arms) in the studies considered in this meta-analysis ranged from 40 to 899.

The pooled mean age of participants was 69.6 years of age, with study-specific means ranging from 55.3 years to 82.3 years. The pooled mean percentage of male participants was 61.7%, but the study by Frea et al. [16] represents an outlier, as 90.5% of participants were male.

Intervention Characteristics

All studies used furosemide as the loop diuretic and compared continuous infusions to intermittent bolus administration. However, the dose of furosemide used and the duration of continuous infusion varied markedly between studies. Whereas most studies specified a dose of furosemide to be infused, or used a specified dose escalation algorithm that was guided by patients’ clinical response to diuretics [29], Felker et al., Frea et al. and Zheng et al. administered “low” or “high” doses to patients, depending on their outpatient diuretic dosages or renal function [16,26,33]. Further, most studies administered the continuous dose of furosemide over 24 hours, but Jaya Shree et al., Llorens et al., and Ragab et al. stipulated hourly furosemide infusion rates [27,28,30]. Protocols for intermittent bolus administration of furosemide also displayed considerable heterogeneity, with variations in the dosages and frequency of bolus administration.

Intervention Characteristics

All studies used furosemide as the loop diuretic and compared continuous infusions to intermittent bolus administration. However, the dose of furosemide used and the duration of continuous infusion varied markedly between studies. Whereas most studies specified a dose of furosemide to be infused, or used a specified dose escalation algorithm that was guided by patients’ clinical response to diuretics [29], Felker et al., Frea et al., and Zheng et al. administered “low” or “high” doses to patients, depending on their outpatient diuretic dosages or renal function [16,26,33]. Further, most studies administered the continuous dose of furosemide over 24 hours, but Jaya Shree et al., Llorens et al., and Ragab et al. stipulated hourly furosemide infusion rates [27,28,30]. Protocols for intermittent bolus administration of furosemide also displayed considerable heterogeneity, with variations in the dosages and frequency of bolus administration.

Outcome Characteristics

Definitions of WRF and AKI were largely consistent across the studies that reported these outcomes [16,26,28-30,33], with most defining them as an increase in sCr of more than 0.3 mg/dL or a urine output of less than 0.5 mL/kg/hour for at least six hours. The exception was the study by Zheng et al. [33], which did not specify a definition for WRF.

Risk of Bias

**Figure 2:**
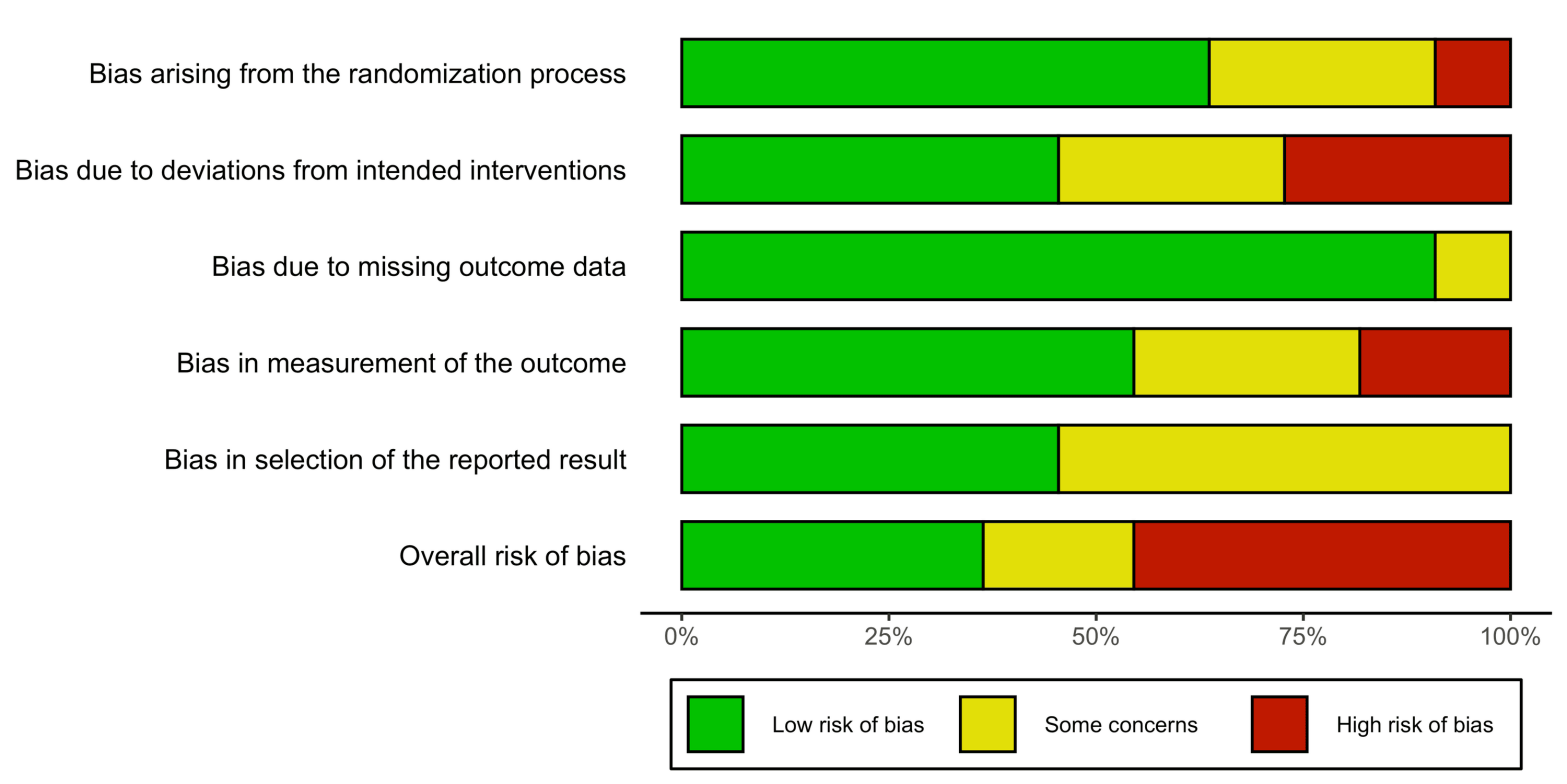
Risk of bias summary bar chart

Table 2 shows the risk of bias assessments by domain, and Figure 2 presents a summary of these assessments.

**Table 2:** Risk of bias assessments of included studies

Study ID	Bias due to randomization (selection bias)	Bias due to deviations from the intended interventions (performance bias)	Bias due to missing data (attrition bias)	Bias due to outcome measurement (detection bias)	Bias due to selection of the reported result (reporting bias)	Overall risk of bias
Frea et al. (2020) [16]	Low	Low	Low	Low	Low	Low
Khan et al. (2024) [17]	Low	High	Low	High	Some concerns	High
Allen et al. (2010) [25]	Low	Low	Low	Low	Low	Low
Felker et al. (2011) [26]	Low	Low	Low	Low	Low	Low
Jaya Shree et al. (2021) [27]	High	High	Some concerns	High	Some concerns	High
Llorens et al. (2014) [28]	Low	High	Low	Low	Some concerns	Some concerns
Palazzuoli et al. (2014) [29]	Low	Low	Low	Low	Low	Low
Ragab et al. (2018) [30]	Some concerns	Some concerns	Low	Some concerns	Some concerns	High
Shah et al. (2014) [31]	Some concerns	Low	Low	Some concerns	Some concerns	High
Yayla et al. (2015) [32]	Some concerns	Some concerns	Low	Some concerns	Some concerns	High
Zheng et al. (2021) [33]	Low	Some concerns	Low	Low	Low	Some concerns

Of the 11 studies included in this meta-analysis, four studies were deemed to have a low overall risk of bias [16,25,26,29], two studies were deemed to have some concerns regarding their overall risk of bias [28,33], and five studies were deemed to have a high overall risk of bias [17,27,30-32]. Given this distribution of bias, the overall certainty of pooled estimates obtained through this meta-analysis is degraded, and it strongly suggested that further well-conducted, adequately concealed RCTs are required before more definitive conclusions can be made.

Quantitative synthesis

WRF

**Figure 3:**
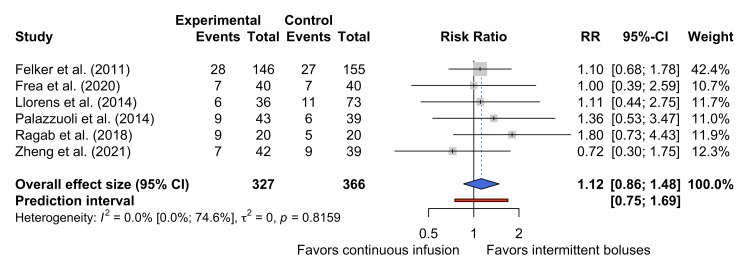
Forest plot of WRF WRF, worsening renal function [16,26,28-30,33]

Six studies reported on the primary outcome of the incidence of WRF in patients receiving continuous infusions and intermittent boluses of loop diuretics [16,26,28-30,33]. Figure 3 shows the pooled data for the 693 participants represented by these studies. This analysis suggests that there was no statistically significant difference in the incidence of WRF between the two dosing strategies (RR 1.12, 95% CI 0.86-1.48).

The study by Felker et al. was the largest contributor to the pooled effect [26], but predated the 2012 KDIGO definition of AKI [20]. However, the definition of WRF used by Felker et al. (sCr rise > 0.3 mg dL⁻¹ within 72 hours) is comparable to the KDIGO definition of AKI as a rise in sCr of more than 0.3 mg dL⁻¹ within 48 hours. This suggests that the outcome definitions were sufficiently comparable for pooling. Furthermore, no between-study heterogeneity was detected (τ² = 0; I² = 0.00%, 95% CI 0.00% to 74.6%, p = 0.82), suggesting that it was appropriate to pool the effect sizes of these studies together.

Although one study [26] predated the 2012 KDIGO definition of AKI [20], this study by Felker et al. defined WRF as a rise in sCr of more than 0.3mg/dL within 72 hours, which is comparable to the KDIGO definition of AKI as a rise in sCr of more than 0.3mg/dL within 48 hours. No heterogeneity was detected between the studies (τ² = 0; I² = 0, 95% CI 0.00% to 74.6%), suggesting that it was appropriate to pool the effect sizes of these studies together.

Furthermore, as noted above, most studies included in the present quantitative synthesis had concordant definitions of WRF. However, Zheng et al. [33] did not provide a definition for their WRF outcome. While subgroup analyses could be performed to analyze the effect of any variations in definitions, the relatively small number of studies reporting on this outcome means that a meaningful result may not be obtained through subgroup analyses. As such, no such subgroup analysis was performed.

In aggregate, this quantitative synthesis suggests that continuous furosemide infusion does not materially affect the risk of WRF or AKI compared to intermittent bolus injections. While the overall direction of the summary measure nominally favors intermittent bolus administration (RR 1.12), the wide CI suggests that this is not a statistically significant effect. The absence of detectable heterogeneity increases confidence that the observed null effect is not driven by outliers, but the small number of participants, reported events, and trials limits the overall certainty of this observed effect.

Increase in sCr

**Figure 4:**
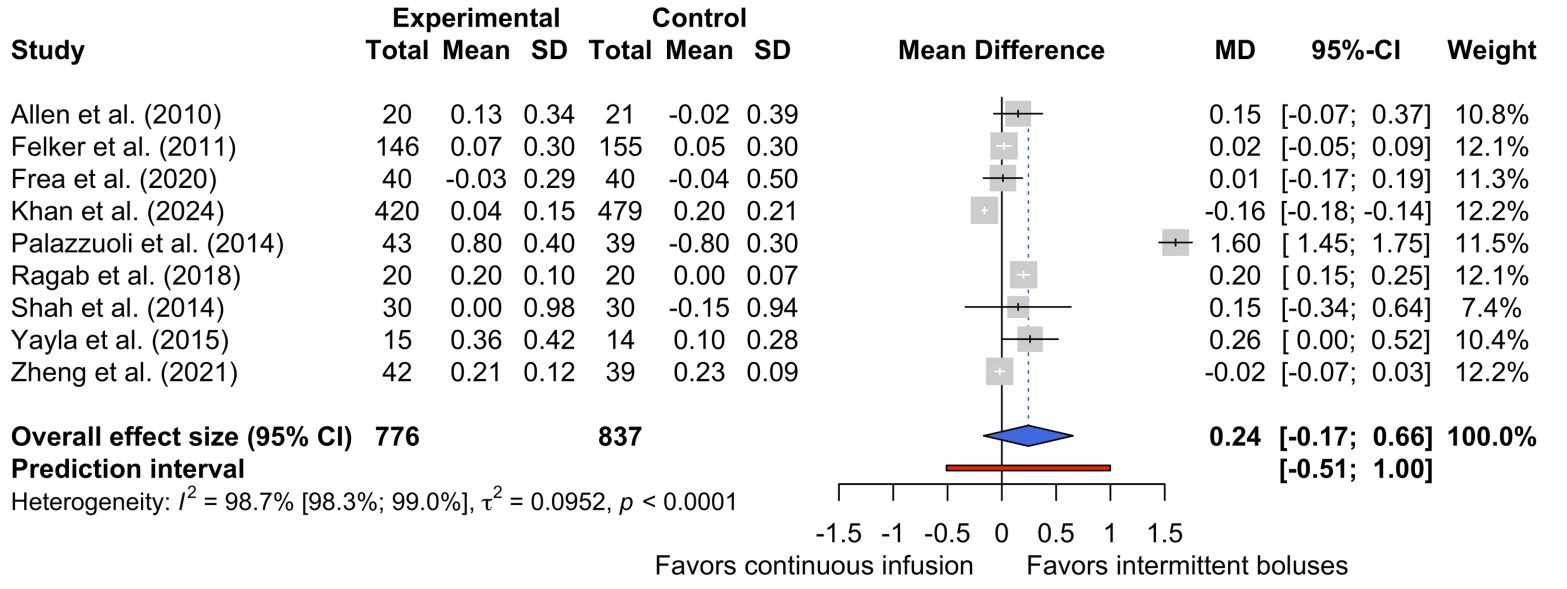
Forest plot of the increase in sCR sCR, serum creatinine [16,17,25,26,29-33]

Nine studies reported the increase in sCr in patients receiving continuous infusions and intermittent boluses of loop diuretics [16,17,25,26,29-33]. Figure 4 shows the pooled data for the 1613 participants represented by these studies and suggests that there is no statistically significant difference in the increase in sCr between the two dosing strategies (MD 0.24 mg dL⁻¹, 95% CI -0.17 mg dL⁻¹ to 0.66 mg dL⁻¹).

There was substantial heterogeneity between these nine studies (τ² = 0.0952; I² = 98.7%, 95% CI 98.3% to 99.0%, p < 0.0001), suggesting that the observed differences are likely to represent genuine differences between trials, rather than sampling error. One important source of heterogeneity was the timing of sCr measurements. While most studies suggested daily measurements of sCr, the timings of the reported changes in sCr varied considerably, meaning that patients in different trials may have noncomparable exposures to furosemide. Other potential sources of variation include the differences in furosemide doses, concomitant nephrotoxic drug use, and differences in participants’ baseline renal function.

Upon inspection of the forest plot in Figure 4, the study by Palazzuoli et al. [29] has a large MD that is not comparable to the other eight studies included in this pooled analysis. This may be because Palazzuoli measured changes in sCr from admission to the end of the protocol (reported as 72 hours), but it is unclear whether this may have been measured to the point of discharge. This study also used the highest dose of furosemide of all studies included in this meta-analysis; such high doses of diuretic may have negatively impacted renal function. While this is a hypothetical explanation for the large MD yielded by Palazzuoli et al. [29], use of a dose-response curve may help to identify whether the large doses of furosemide administered in this study accounted for the unexpected effect on sCR. However, as many studies did not provide an accurate mean value of furosemide administered, it was not possible to draw a dose-response curve to explore this hypothesis.

**Figure 5:**
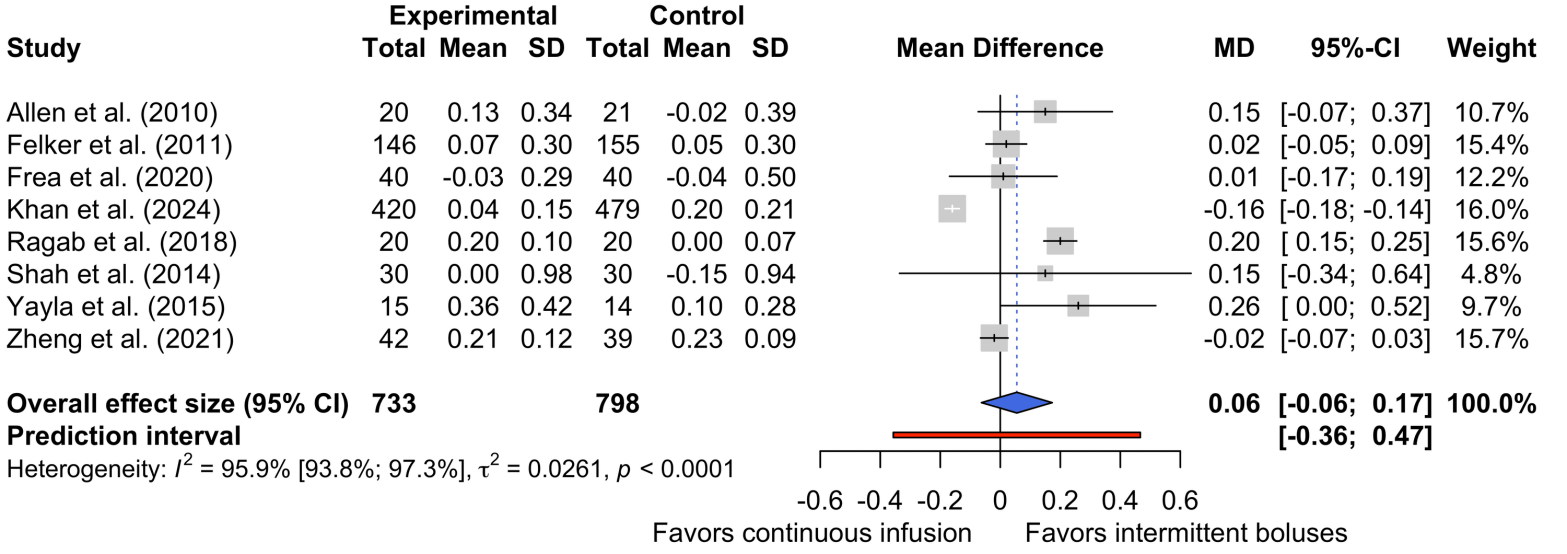
Forest plot of the increase in sCR, excluding Palazzuoli et al. sCR, serum creatinine [16,17,25,26,30-33]

Given its influence, the study by Palazzuoli et al. was treated as an outlier in a repetition of the quantitative synthesis (Figure 5). However, high levels of heterogeneity persisted (τ² = 0.0261; I² = 95.9%, 95% CI 93.8% to 97.3%, p < 0.0001), further demonstrating the extent of differences in study design.

In summary, this quantitative synthesis suggests that continuous furosemide infusion does not significantly increase sCr compared to intermittent bolus injections. If sCr is inferred to be a correlate of renal function, the direction of the MD (0.24) obtained in this quantitative synthesis suggests that intermittent bolus injections may be less harmful to the kidneys than continuous infusions. However, given that the CI straddles the line of no effect and the high levels of heterogeneity in this analysis, this is not a statistically significant or certain effect.

sCR at Discharge

**Figure 6:**
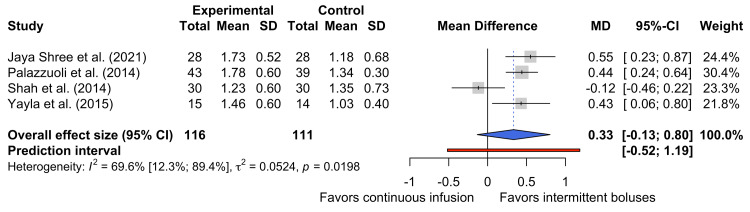
Forest plot of discharge sCr levels sCR, serum creatinine [27,29,31,32]

Four studies reported sCr levels at the point of discharge [27,29,31,32]. Figure 6 shows the pooled data for the 227 participants represented by these studies and suggests that there is no statistically significant difference in the sCr at discharge between the two dosing strategies (MD 0.33 mg dL⁻¹, 95% CI -0.13 mg dL⁻¹ to 0.80 mg dL⁻¹).

There was high heterogeneity between these four studies (τ² = 0.0524; I² = 69.6%, 95% CI 12.3% to 89.4%, p = 0.0198), suggesting that there are likely to be clinical and methodological differences between the studies. Such differences are likely to have arisen from differences in the length of hospitalization and discharge criteria between the studies.

The pooled MD marginally favors intermittent bolus administration, suggesting that patients receiving continuous diuretic infusions may experience higher discharge creatinine levels and worse renal function. However, this is not a statistically significant finding, and the high heterogeneity associated with this pooled effect estimate precludes definitive conclusions regarding the impact of diuretic administration on discharge creatinine levels being made.

Serum BUN at Discharge

**Figure 7:**
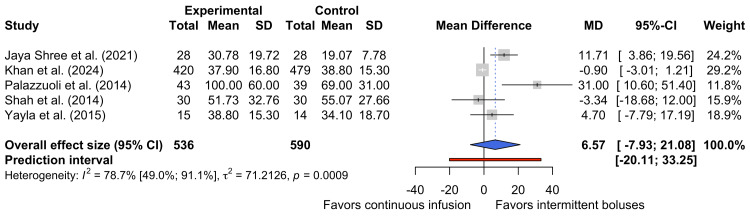
Forest plot of discharge BUN levels BUN, blood urea nitrogen [17,27,29,31,32]

Five studies reported serum BUN concentrations at the point of discharge [17,27,29,31,32]. Figure 7 shows the pooled data for the 1126 participants represented by these studies. This quantitative synthesis suggests that no statistically significant difference in serum BUN concentrations at discharge between the two dosing strategies (MD 6.57 mg dL⁻¹, 95% CI -7.93 mg dL⁻¹ to 21.08 mg dL⁻¹).

There was significant heterogeneity between the five studies (τ² = 71.2126; I² = 78.7%, 95% CI 49.0% to 91.1%, p = 0.0009), suggesting that almost 80% of the observed variance is reflective of differences between studies. Such heterogeneity may have arisen from differences in discharge criteria and dosages of furosemide between studies. 

The pooled MD of this quantitative synthesis marginally favors intermittent bolus administration, as patients receiving continuous infusions may experience higher BUN levels at discharge than those receiving intermittent boluses. However, this is neither a statistically significant nor a certain finding, as the pooled effect size crosses the line of no effect and is limited by significant heterogeneity.

Length of Hospitalization

**Figure 8:**
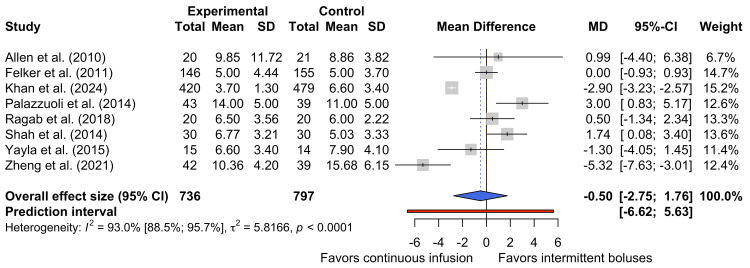
Forest plot of length of hospitalization [17,25,26,29-33]

Eight studies reported the length of hospitalization [17,25,26,29-33]. Figure 8 shows the pooled data for the 1533 participants represented by these studies. The pooled effect suggests that there is no statistically significant difference in the length of hospitalization between patients assigned to continuous infusion groups and those assigned to intermittent bolus groups (MD -0.50 days, 95% CI -2.75 days to 1.76 days).

There was substantial heterogeneity between the studies included in this analysis (τ² = 5.81166; I² = 93.0%, 95% CI 88.5% to 95.7%, p < 0.0001). Such heterogeneity is likely to have arisen from the evident differences in the dose of diuretic administration between studies (as noted in Table 1), differences in discharge criteria, and differences in the settings in which these studies took place. As demonstrated in Table 1, this meta-analysis included studies conducted across a range of countries, including Canada, China, Egypt, India, Italy, Pakistan, Spain, Türkiye, and the USA. The differences in healthcare resources and logistics in these nations may mean that discharge criteria also differ, leading to underlying differences that may explain some of the heterogeneity observed in this analysis.

In aggregate, the evidence considered in this quantitative synthesis does not support the preferential use of continuous furosemide infusions or intermittent bolus administration as a means to reduce the length of hospitalizations in patients with ADHF. While the overall pooled effect is marginally in favor of continuous diuretic administration, the 95% CI for this estimate crosses the line of no effect, suggesting that the observed differences in length of hospitalization were not statistically significant.

Risks of Bias Across Studies

While the protocol for this study envisaged the construction of a funnel plot to evaluate the extent of publication bias, only six studies reported on the primary outcome. In view of this, a funnel plot or alternative metrics of evaluation could not be constructed; current methodological guidance suggests that the use of funnel plot asymmetry to evaluate publication bias is not recommended when there are fewer than 10 studies, as this may result in unacceptably high risks of false-positive or false-negative conclusions [34].

Post Hoc Analysis: TSA

To assess the robustness and sufficiency of the accumulated evidence for the primary outcome of WRF, a TSA was performed in R as a post hoc sensitivity assessment [35]. Two-sided testing was performed, with type I error (α) set at 0.05, type II error (β) set at 0.20 (power = 0.80), and a relative risk reduction threshold of 20% (m = 0.8). The O’Brien-Fleming alpha-spending [36] and Pocock beta-spending functions [37] were used under a random-effects model.

**Figure 9:**
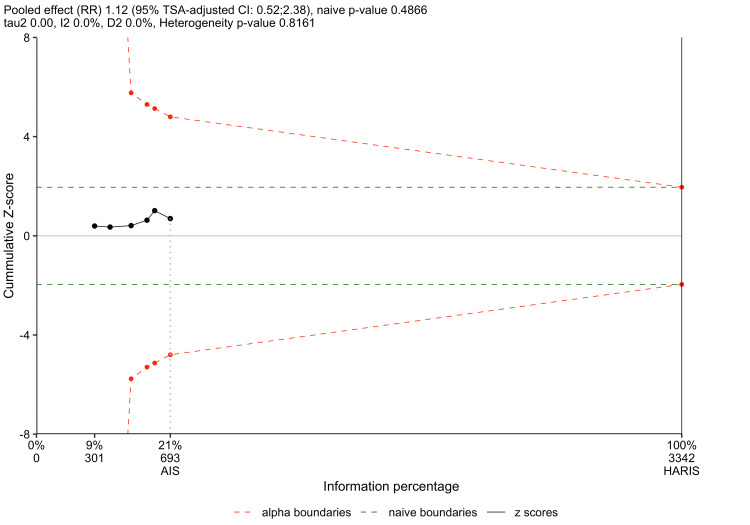
TSA plot TSA, trial sequential analysis

The TSA plot is depicted in Figure 9. The cumulative Z-curve did not cross either the conventional significance boundary or the TSA-adjusted monitoring boundaries, further reinforcing the nonstatistical significance of the findings in this meta-analysis. The calculated required information size (RIS) was 3342 participants. Given that the accrued information size was 693 participants in this meta-analysis, this TSA indicates that current evidence is insufficient to draw definitive conclusions regarding the effect of continuous versus intermittent IV furosemide on the incidence of WRF in ADHF. The small number of trials on the present subject matter means that the number of included participants is too small to meet the RIS for TSA. This somewhat restricts the utility of the TSA in drawing inferences on the sufficiency of accumulated evidence for the primary outcome.

Discussion

Continuous infusions of loop diuretics have been demonstrated to result in more constant plasma diuretic concentrations and avoid the peak-trough fluctuations associated with intermittent bolus injection [12]. This, in turn, is thought to result in reduced neurohormonal stimulation and reduced renal injury [38]. Based on these physiological effects, and in the absence of international guidelines on the matter, treating physicians may choose to administer loop diuretics via continuous infusions (as opposed to intermittent boluses) in patients with ADHF to minimize the risk of renal injury.

Key Findings

This meta-analysis identified 11 RCTs, enrolling a total of 1784 patients, and found no compelling evidence that continuous infusions of loop diuretics are superior to intermittent IV boluses in terms of renal outcomes. The findings of the TSA further indicate that, despite an evolving body of comparative data, the current evidence base remains underpowered and inconclusive for detecting clinically significant differences in the risk of WRF between diuretic strategies. This reinforces the need for further high-quality randomized trials explicitly powered to evaluate renal endpoints in this context. 

There was no statistically significant difference in the incidence of WRF between the methods of diuretic administration. Similarly, there was no statistically significant difference in the increases in sCr, levels of sCr at discharge, levels of serum BUN at discharge, and length of hospitalization between patients receiving continuous infusions of loop diuretics and those receiving intermittent infusions. Although previous meta-analyses have not compared the incidence of WRF between these methods of diuretic administration, our findings align with earlier meta-analyses, which noted no significant difference in the length of hospitalization [14,15,22] or in plasma creatinine concentrations at discharge [15]. By uniquely exploring the specific renal outcome of WRF and considering two RCTs [16,17] not considered in the previous meta-analysis by Karedath et al. [14], we broaden the evidence base and suggest that continuous infusions of loop diuretics do not offer additional renal protection over intermittent boluses in the treatment of ADHF.

Heterogeneity

However, it must be noted that these outcomes were subject to high levels of heterogeneity, suggesting that findings must be interpreted with caution and limiting the generalizability of the observed pooled effects. Such heterogeneity may, indeed, explain why the theorized physiological benefits of continuous loop diuretic infusions do not translate into measurable clinical benefits.

One source of heterogeneity that may confound the observed result is the disparity in the dosages of furosemide administered. High doses of furosemide are more likely to result in more severe renal insults than lower doses, as observed by Felker et al. [26], who noted that individuals assigned to the “high” dose arm (2.5 times the outpatient diuretic dose) had a higher frequency of a rise in sCr of more than 0.3 mg dL⁻¹ compared to those assigned to the “low” dose arm. Higher doses of furosemide may result in higher plasma drug concentrations and WRF [39], meaning that the diuretic dose is likely to independently affect the incidence of WRF regardless of the method of administration. A future meta-analysis may consider subgroup analyses by diuretic doses, but further RCTs are required, as the Cochrane Handbook for Systematic Reviews of Interventions recommends at least 10 studies per characteristic before performing subgroup analyses.

The RCTs included in this trial were also subject to varying risks of bias. Of the 11 included studies, five were deemed to have a high risk of bias [17,27,30-32]. These biases must be recognized when interpreting the pooled results of this study. While meta-analyses generally offer the highest level of evidence, the validity of their conclusions is dependent on the methodological quality of included studies. Many studies also had inconsistent definitions of renal endpoints, further impinging on their validity. The inclusion of studies with such high risks of bias may limit the internal validity of our findings and reinforces the need for further high-quality RCTs in this field.

Strengths and Limitations

This meta-analysis used rigorous methods that are in keeping with PRISMA guidelines and only considered RCTs, eliminating crossover trials and observational studies. However, this study is subject to limitations. Firstly, there was substantial variation in the patient characteristics and interventions among the included RCTs, contributing to a high level of heterogeneity and minimizing the generalizability of findings. The generalizability of findings is further limited by the lack of large, international, multicenter RCTs on this subject. This means that the observed findings of this study may not necessarily be applicable across different countries and healthcare systems and highlights the need for further international, large-scale trials on this matter. Secondly, the primary outcome was limited by a relatively small event size, as there were only 131 instances of WRF among the 693 participants. This results in a potential for rare, but clinically significant differences that may remain undetected. The small number of studies reporting on the primary outcome also meant that subgroup analyses (including by risk of bias) could not be performed, preventing a thorough appraisal of the effects of bias on this study. Thirdly, this meta-analysis was limited by the fact that the search strategy was restricted to the PubMed database and that studies from the grey literature, conference abstracts, and those published in a language other than English were excluded. These criteria may have introduced a selection bias into the results of the present analysis. The present analysis was also not registered with PROSPERO, but to minimize the risk of a selective reporting bias, a pre-specified study protocol was drafted and disseminated to authors prior to the commencement of this study.

Future Research

In the future, large, well-powered, international, multicenter RCTs with standardized dosing algorithms and precisely defined renal end points are required to help definitively determine whether continuous infusions of loop diuretics are superior to intermittent bolus injections in preserving renal function among patients with ADHF. In view of the high heterogeneity in dosing algorithms and definitions of renal outcomes among the trials included in this study, conducting further RCTs on this matter (as specified) will increase the robustness of evidence and allow for a fairer comparison of the effects of continuous and intermittent IV administration of furosemide in the treatment of ADHF across different healthcare systems and settings.

## Conclusions

In this meta-analysis of 11 RCTs, continuous furosemide infusions did not have a statistically significant effect in reducing the incidence of WRF compared to intermittent bolus administration. There was also no statistically significant difference in surrogate markers of renal function or length of hospitalization between the two diuretic strategies. This may support the current practice of physician discretion in determining whether to administer loop diuretics by continuous infusion or intermittent IV bolus administration in patients with ADHF; such decisions may be augmented based on patient- and institution-specific factors. However, given the heterogeneity and risks of bias among the included studies, these findings must be interpreted with caution, and the certainty of such evidence is low. Further, high-quality, standardized RCTs remain necessary before definitive guideline recommendations on the renal safety of these diuretic administration strategies can be issued, though there are likely to be logistical and ethical hurdles associated with conducting such studies.

## Appendices

Appendix A: PubMed search strategy

(heart failure OR cardiac failure OR HF OR ADHF OR decompensated heart failure OR congestive heart failure OR left ventricular failure) AND (diuretic OR diuretics OR loop diuretic OR loop diuretics OR furosemide OR frusemide OR bumetanide OR torsemide OR torasemide OR water pill OR Lasix OR Demadex) AND (continuous OR continuous administration OR continuous infusion OR infusion OR intravenous OR IV OR IV infusion OR drip OR bolus OR intermittent OR injection) AND (renal OR kidney OR renal function OR kidney function OR renal insufficiency OR kidney injury OR renal injury OR acute kidney injury OR AKI OR renal failure OR kidney failure OR acute renal failure OR nephrotoxic* OR creatinine OR serum creatinine OR eGFR OR glomerular filtration rate OR estimated glomerular filtration rate OR cystatin C OR creatinine clearance OR CrCl OR urea OR BUN)
